# Short-Term Photovoltaic Power Forecasting Based on Historical Information and Deep Learning Methods

**DOI:** 10.3390/s22249630

**Published:** 2022-12-08

**Authors:** Xianchao Guo, Yuchang Mo, Ke Yan

**Affiliations:** 1 Fujian Province University Key Laboratory of Computational Science, Huaqiao University, Quanzhou 362021, China; 2 Department of the Built Environment, College of Design and Engineering, National University of Singapore, Singapore 117566, Singapore

**Keywords:** Bayesian optimization algorithm, bidirectional long short-term memory, deep learning, singular spectrum analysis, short-term photovoltaic power forecasting

## Abstract

The accurate prediction of photovoltaic (PV) power is essential for planning power systems and constructing intelligent grids. However, this has become difficult due to the intermittency and instability of PV power data. This paper introduces a deep learning framework based on 7.5 min-ahead and 15 min-ahead approaches to predict short-term PV power. Specifically, we propose a hybrid model based on singular spectrum analysis (SSA) and bidirectional long short-term memory (BiLSTM) networks with the Bayesian optimization (BO) algorithm. To begin, the SSA decomposes the PV power series into several sub-signals. Then, the BO algorithm automatically adjusts hyperparameters for the deep neural network architecture. Following that, parallel BiLSTM networks predict the value of each component. Finally, the prediction of the sub-signals is summed to generate the final prediction results. The performance of the proposed model is investigated using two datasets collected from real-world rooftop stations in eastern China. The 7.5 min-ahead predictions generated by the proposed model can reduce up to 380.51% error, and the 15 min-ahead predictions decrease by up to 296.01% error. The experimental results demonstrate the superiority of the proposed model in comparison to other forecasting methods.

## 1. Introduction

With the rapid development of modern society, global energy production and demand have increased dramatically [1]. However, traditional energy stocks are limited, and carbon dioxide emissions during combustion can cause environmental problems, including global warming and the greenhouse effect. As a result, the adjustment of the energy structure and the development and utilization of new energy sources have gradually attracted the attention of all countries in the world [2]. Solar energy is a clean, green, sustainable, and renewable energy source. In the case of falling costs, the penetration rate of solar energy in the energy market is gradually increasing [3].

In recent years, solar PV power plants have been widely used because of their intense power generation capacity. However, the random, volatile, and intermittent nature of PV power data causes the grid system to face significant issues such as stability, reliability, and power balance. Accurate short-term PV power forecasting plays a vital role in the security, stability, and economic operation of the power grid and the dispatching plan of PV power systems [4]. Therefore, numerous studies have been conducted on PV power prediction, with a brief survey provided in the rest.

In [5], a review of PV power forecasting models is given. PV power forecasting techniques mainly include persistence forecast methods, physical models, statistical techniques, machine learning forecast techniques, and deep learning strategies. Persistence models suggest that today’s solar irradiance is similar to the day before [6]. However, if weather conditions change considerably, persistence methods often fail. Physical techniques rely on the current weather service data and satellite image data. Aguiar et al. [7] used NWP and satellite data to forecast Spain’s global horizontal irradiance. Moreover, physical models do not require historical PV data but use specific weather characteristics. Similarly, false forecasts occur when the values of weather variables change suddenly. In [8], a step-by-step method based on the autoregressive moving average (ARMA) is proposed for PV power prediction for the next hourly time horizon. However, statistical techniques are limited in handling large amounts of data. Machine learning algorithms are applied to time series data forecasting. Kaushika et al. [9] were the first to apply the feedforward neural network (FFNN) algorithm to the field of PV power prediction using weather information. In [10], a novel model based on neural networks and multilayer perceptrons used a least squares optimization algorithm to predict the next 24 h. Huang et al. [11] proposed a data-driven framework that fuses the spatiotemporal information of target and neighboring PV sites. The multi-step ahead prediction of solar irradiance showed that boosted regression trees are more suitable than the benchmark model.

Various deep learning (DL) methods have recently been introduced for time series data forecasting issues. However, the temporal dependencies in PV power generation data are not considered by artificial neural networks (ANNs). Meanwhile, the advantage of deep learning techniques is that they have a significant capacity for nonlinear data. LSTM has been applied to time series data prediction issues [12], air quality forecasting [13,14], load forecasting [15], and solar irradiation forecasting [16,17], to name a few. Numerous prediction experiments have demonstrated how different data pretreatment techniques significantly boost the neural network model’s capacity for prediction [18]. In [13], a multitask multi-channel nested LSTM (NLSTM) hybrid deep learning framework combining stationary wavelet transform (SWT) was proposed to forecast air quality. In comparison to support vector machines (SVM), Yan et al. [19] asserted that LSTM neural networks perform better at capturing the dependencies between data samples. They proposed a novel DL forecasting framework combining cutting-edge LSTM and the widely used SWT. In another study, Yan et al. [20] applied a multichannel extension LSTM model to forecast energy consumption from data collected in London, UK. Jin et al. [21] proposed a deep learning model based on singular spectrum analysis and LSTM for 5 min energy consumption forecasting. In [22], a support vector regression (SVR) model with a convolutional neural network gated recurrent unit (CNNGRU) network was designed. The SSA algorithm was used to decompose the raw wind speed data. The proposed method was evaluated using three verification datasets. Barbieri et al. [23] developed a novel method for predicting very short-term PV output using satellite and sky imaging. Furthermore, it implies that hybrid models can improve forecast accuracy. Yao et al. [4] proposed an innovative deep learning-based forecasting system incorporating an improved U-net and an encoder–decoder architecture. Several experiments are provided to discuss the ideal framework configuration and structure. It achieved maximum error reductions of 4.561% and 3.55% in terms of *RMSE* and *MAE*. Ren et al. [24] applied an accurate intra-hour PV power forecasting model with the quad-kernel deep convolutional neural network (CNN) for case studies from a 26.52 kW PV plant. Yan et al. [25] developed a CNN-LSTM integrated model for short-term load forecasting at multi-step predicted horizons. Zang et al. [26] created a hybrid model to predict PV power using the two-dimensional CNN method, resulting in a lower forecast error across multiple evaluation criteria. In another study [27], PV power generation was forecasted for time horizons ranging from 7.5 min-ahead to 60 min-ahead by combining the LSTM model with the attention mechanism. The attention mechanism adaptively focuses on more significant input features when taking into account how temperature data affect PV power generation. The prediction effect outperforms the comparison model in each time domain. In [28], a hybrid LSTM convolutional model was presented to forecast the PV power 5 min in advance. The fusion order influences the model’s prediction accuracy and time consumption significantly. The LSTM-CNN model outperforms CNN-LSTM in terms of performance and computational time. In [29], an effective PV power forecasting framework was developed based on wavelet analysis and LSTM with a stochastic differential equation to provide accurate prediction information in different seasons. Pi et al. [16] used multichannel wavelet transform combining CNN and BiLSTM networks to forecast solar irradiance under different time horizons. Shah et al. [30] forecasted electricity price and demand successfully, and the Diebold and Mariano test was used to determine the statistical significance of differences in model performance. According to the current literature, the data decomposition algorithm with LSTM offers the excellent capability to handle volatile and intermittent data.

As might be deduced from the justifications above, PV power forecasting has drawn academic attention recently. However, deep neural network (DNN) creation is a laborious iterative process that involves both technical knowledge and trial-and-error experience. Additionally, tuning the DNN hyperparameters is crucial since the network performance’s effectiveness is highly related to its architecture [17]. It can be inferred from studying prior research in the area of PV power forecasting that most of these studies manually constructed deep structures through trial and error. This process is quite difficult computationally due to the long execution time of a tailored architecture. Thus, this paper proposes a method to automatically produce accurate predictions of PV power without manually tuning the deep learning architectures [31,32]. Zhou et al. [33] developed a PV forecasting framework with a signal decomposition technique and a multi-objective chameleon swarm algorithm to predict short-term PV power. The R-square scores obtained from Safi-Morocco are 0.995, 0.993, and 0.995, respectively. The performance of the time series can be improved based on the optimization algorithm. To this end, a combinatorial model that combines the SSA and optimized architecture of BiLSTM with BO is proposed to create a highly reliable model. In conclusion, the main contributions of this paper are as follows:

(1) **A neural network structure composed of several parallel BiLSTM neural networks.** Each BiLSTM is used to train the corresponding sub-signal produced by the SSA. The integrated approach enables more precise prediction by processing multiple sub-signals, potentially improving the final prediction accuracy.
(2) **Applying the Bayesian optimization algorithm in PV power prediction.** Bayesian optimization is considered to search hyperparameters for LSTM. The proposed Bayesian optimization algorithm enhances the prediction performance of LSTM.
(3) **A novel artificial intelligence forecasting framework that combines SSA and parallel BiLSTM neural networks.** The SSA is intended for denoising and feature extraction to improve prediction performance. The SSA outputs are fed in parallel to a series of BiLSTM neural networks.
(4) **A comprehensive comparative study.** We compare the proposed SSA-BO-BiLSTM with cutting-edge technologies, including machine learning methods and various extensions of LSTM. The experimental results show that the proposed model outperforms the existing models in terms of effectiveness and efficiency.

The remainder of this paper is organized as follows: Section 2 describes the theoretical methods. In Section 3, the results of the numerical experiments are presented. In Section 4, a discussion of the experimental results is provided. Finally, this paper is concluded in Section 5.

## 2. Methodology

This section illustrates the details of the proposed PV power hybrid forecasting framework. Firstly, the PV power series is decomposed into stationary sub-signals with a singular spectrum analysis algorithm [34]. Then, the parameters of the BiLSTM model are tuned using the Bayesian optimization algorithm. Subsequently, the BiLSTM neural network is connected to each sub-signal for forecasting. Finally, we presented the overall architecture of the proposed model.

### 2.1. Singular Spectrum Analysis

**Figure 1:**
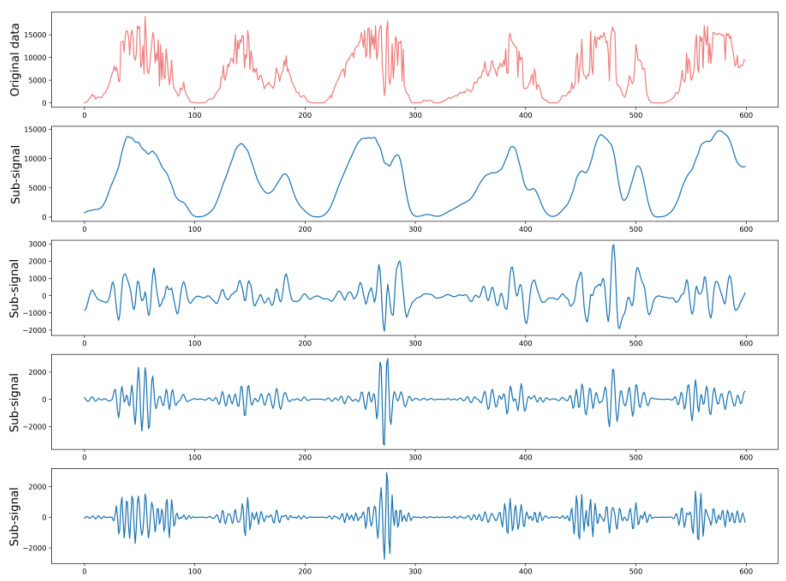
SSA decomposed several subsequences of the original time series.

Singular spectrum analysis is a nonparametric time series decomposition method that combines the time and frequency domains [22,35]. SSA can extract the intrinsic driving characteristics of sequence fluctuations and denoise original PV power data. The SSA technique is utilized to preprocess data by signal decomposition. Light coral represents the original signal, and blue represents the decomposition signal, as shown in Figure 1. Compared to the initial time series data, the decomposed sub-signals are more regular, effectively reducing the difficulty of prediction. Such a strategy demonstrates its potential to improve the forecasting performance of BiLSTM neural networks.

SSA includes decomposition and reconstruction stages [36]. The decomposition stage includes two sub-processes of embedding and singular value decomposition. The reconstruction stage includes two sub-processes of grouping and diagonal averaging. The detailed steps of the SSA can be explained in the following subsections:

#### 2.1.1. Embedding

Assuming the original time series with *F* observations, the original one-dimensional *Y* = [*y*_1_, *y*_2_, …, *y_F_*]^T^ is converted into a multi-dimensional time series by the sliding window method with embedded dimension *L*, then there is a trajectory matrix *X* as follows (1):

(1) $$X=[x_{1},x_{2},\cdots ,x_{K}]=\left(\begin{array}{cccc} y_{1} & y_{2} & \cdots & y_{K}\\ y_{2} & y_{3} & \cdots & y_{K+1}\\ \vdots & \vdots & & \vdots \\ y_{L} & y_{L+1} & \cdots & y_{F} \end{array}\right),$$

where *K* = *F* − *L* + 1, 2 ≤ *L* ≤ *F*/2. *i* = 1, 2, …, *K* is called the *L-lagged* vector.

#### 2.1.2. Singular Value Decomposition

Let *S* = *XX^T^*. Denote *λ*_1_, *λ*_2_, …, *λ_L_* as the values of the matrix *S*, and the corresponding eigenvector is *U*_1_, *U*_2_, …, *U_L_*. The singular value $\sqrt{\lambda_{i}}$ denoises the original signal. Additionally, matrix *X* is decomposed using (2):

(2) $$X=U\Sigma V^{T}=\sum_{i=1}^{d}\sqrt{\lambda_{i}}U_{i}V_{i}{}^{T},\;d=max\{i,\lambda_{i}>0\},$$

where *U*, *V*, and Σ are the left matrix, right matrix, and diagonal matrix, respectively.

#### 2.1.3. Sequence Grouping

Divide index *I* = (1, 2, …, *d*) into *p* groups *I*_1_, *I*_2_, …, *I_P_* with no intersection. Define *I* = (*i*_1_, *i*_2_, …, *i_m_*) for each group, and the corresponding result matrix is *X_I_* = *X_i_*_1_ + *X_i_*_2_ + ⋯ + *X_im_*. Trajectory matrix *X* is converted by using the following (3):

(3) $$X=X_{I_{1}}+X_{I_{2}}+\cdots +X_{Ip}.$$

#### 2.1.4. Diagonal Averaging

Let *M* = *L* × *K*, *M_ij_* is the element of matrix *M*, where 1 ≤ *i* ≤ *L*, 1 ≤ *j* ≤ *K*. The diagonal average converts *M* into a sequence {*M*_1_, *M*_2_, …, *M_F_*}, given as (4):

(4) $$y_{k}=\{\begin{array}{cc} \frac{1}{k}\sum_{q=1}^{k+1}M_{q,k-q+1}^{*} & 1\leq k\leq L^{*},\\ \begin{array}{c} \frac{1}{L^{*}}\sum_{q=1}^{L^{*}}M_{q,k-q+1}^{*}\\ \frac{1}{F-k+1}\sum_{q=k-K^{*}+1}^{N-K^{*}+1}M_{q,k-q+1}^{*} \end{array} & \begin{array}{l} L^{*}\leq k\leq K^{*},\\ K^{*}\leq k\leq F, \end{array} \end{array}$$

where L* = *min*(*L*, *K*), K* = *max*(*L*, *K*). The original sequence *y_i_* can be decomposed as (5):

(5) $$y_{i}=\sum_{k=1}^{L}y_{i}^{k},\;i=1,\;2,\;\cdots ,F.$$

### 2.2. Bayesian Optimization

Hyperparameter optimization enhances the performance of deep neural networks. The parameter tuning methods include experimental trial-and-error methods, a grid search, and a random search. However, these methods have some shortcomings. For example, the experimental trial-and-error method is time-consuming and not always optimal for the performance of the network. Grid search is inefficient even with parallel computing. Past work has shown that random search is more efficient than grid search [32], but the problem is that it is easy to miss accurate solutions. In contrast to traditional methods, Bayesian optimization generates every guess based on previous training results. It requires fewer iterations and has a higher computational speed. More importantly, Bayesian optimization remains robust when dealing with nonconvex problems and is less likely to fall into local optima.

Bayesian optimization uses the probabilistic model surrogate function to fit the objective function [37,38]. Then, the following hyperparameter combination is chosen to sample based on the posterior probability distribution. Let *α* = *α*_1_, *α*_2_, …, *α_n_* be a set of hyperparameter combinations, *f* (*α*) is the objective function of the hyperparameter *α*. The principle of Bayesian optimization is to find that *α* belongs to *U* such that:

(6) $$\alpha^{*}=\arg\underset{\alpha \in U}{\max}f(\alpha ).$$

In other words, the algorithm aims to find the optimal parameter set by minimizing the loss value *L* while making the generalization error as small as possible. The steps of Bayesian optimization are as follows:

(1) A Gaussian process (GP) calculates the posterior probability distribution. The GP is a set of random variables in which any linear combination of finite samples has a joint Gaussian distribution. GP is represented by the mean function *m* and the covariance matrix function *k* as follows in (7):

(7) f∈GP(m,k).

(2) Apply the upper confidence bound (UCB) method to determine the next sample location. μ represents development, model uncertainty σ represents exploration, and *k* is the hyperparameter that can control the focus of development and exploration. Avoid local optima by making a trade-off between exploration and exploitation.

(8) UCB(x)=μ(x)+kσ(x).

(3) Output suitable hyperparameters.

### 2.3. BiLSTM Neural Network

In recurrent neural networks, the influence of long-term historical information on the output at the current moment decreases with the passage of the sequence, and the problem of gradient disappearance occurs. The hidden layer of the LSTM replaces simple neurons with complex memory modules and retains historical information more reliably [12].

**Figure 2:**
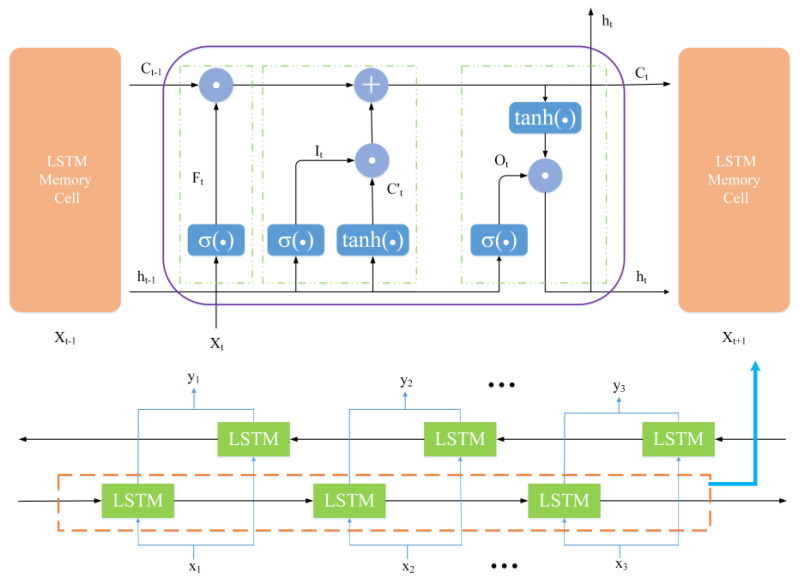
The internal structure of the BiLSTM unit.

Figure 2 shows the memory module structure of the BiLSTM network. The memory module consists of forget gate *F_t_*, input gate *I_t_*, and output gate *O_t_*. *F_t_* (9) decides which information to discard at the current moment through the sigmoid function. *I_t_* (10) decides which information needs to be updated through the sigmoid layer, $C_{t}^{\prime }$ (11) generates alternative cell state new information through the *tanh* layer. After the forgetting and input links are completed, the long-term memory is updated. *O_t_* (13) determines what information will be output to the next layer of LSTM. At the current time *t*, the final output *h_t_* (14) is defined by the current cell state *C_t_* (12) and the output gate *O_t_*.

(9) $$F_{t}=\sigma (W_{F}\cdot \;[h_{t-1},x_{t}]+b_{F}),$$

(10) $$I_{t}=\sigma (W_{I}\cdot \;[h_{t-1},x_{t}]+b_{I}),$$

(11) $$C_{t}^{\prime }=\tanh(W_{C}\cdot \;[h_{t-1},x_{t}]+b_{C}),$$

(12) $$C_{t}=F_{t}⊙C_{t-1}+I_{t}⊙C_{t}^{\prime },$$

(13) $$O_{t}=\sigma (W_{O}\cdot \;[h_{t-1},x_{t}]+b_{O}),$$

(14) $$h_{t}=O_{t}⊙\tanh(C_{t}),$$

where *W* denotes the corresponding weight, and *b* represents the corresponding bias values. [,] is the joining of two matrices, ⊙ is the multiplication of the related elements of the matrix, and ⊕ is the matrix addition operator. *σ* and *tanh* are activation functions.

One-way LSTM cannot obtain past and future information at the same time. As shown in Figure 2, BiLSTM inputs the time series forwards and reverses to two LSTM memory modules. The outputs of the two modules are jointly sent to the output layer, which effectively solves the information timing problem.

### 2.4. Hybrid Forecasting Model

**Figure 3:**
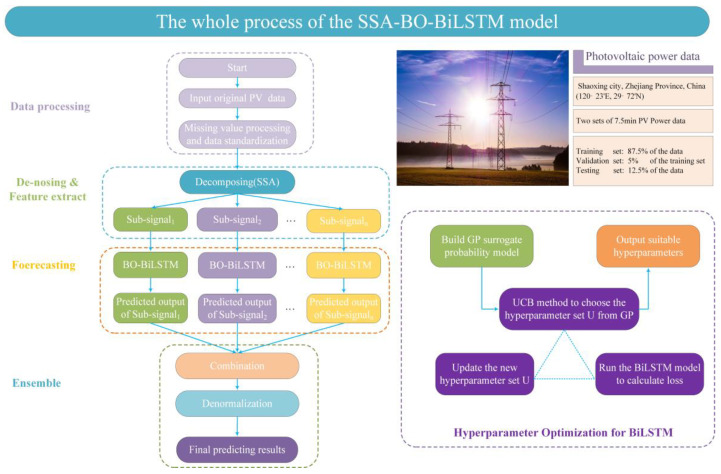
Illustration of the SSA-BO-BiLSTM prediction strategy.

In this paper, an artificial intelligence (AI) model for short-term PV power forecasting is proposed. Figure 3 shows the details of the AI model architecture. The whole process can be described in five steps as given below:

Step 1: The original data should be preprocessed before being input into the model. Firstly, the Augment Dickey–Fuller (ADF) test is used to determine whether the data are stationary. If the result is refused, differential processing is needed. Then, missing values are handled. We follow the principle of taking the mean of the previous and next rows of outliers. Last but not least, we utilize the Z-Score to standardize the raw PV power time series with an average value of 0 and a standard deviation of 1.
Step 2: The processed data are decomposed into four sub-signals by implementing the SSA process. These decomposed signals exhibit much more stable behavior. The sub-signals are split training sets, validation sets, and testing sets. Take the data of 87.5% as the training set, the training set of 5% as the validation set, and the data of 12.5% as the testing set.
Step 3: The Bayesian optimization algorithm is used to select appropriate parameters. It is noteworthy that parameters are an important issue in LSTM, which need to be given in advance. In this study, LSTM is trained using Bayesian optimization to enhance the predictor’s performance.
Step 4: Forecasting each sub-signal with BiLSTM. The sub-signals are the input of the parallel BiLSTM neural network. After training, the testing dataset is utilized to obtain the predicted results. Subsequently, denormalization of the forecasting results is performed.
Step 5: Perform assessment functions to measure the prediction performance of PV power in terms of goodness of fit and error severity.

## 3. Experimental Process and Results

### 3.1. Data Description and Analysis

The original PV power generation data are collected from July to September 2017 and July to November 2016 from a roof PV power plant in eastern China. The dataset [29] is accessible from the Power and Energy datasets of IEEE DataPort. Each dataset is 7.5 min intervals of PV power. The measurement of the sensor data lasts approximately 12 h from sunrise to night. Table 1 provides the statistical information for each dataset.

For PV power dataset 1, 7000 data samples and 1000 data samples are used for training and testing, respectively. Then, 350 validation data are extracted from the training data. Similarly, PV power dataset 2 is divided into 11,200 data samples, 1600 data samples, and 560 data samples as the training test, testing test, and validation test, respectively.

**Figure 4:**
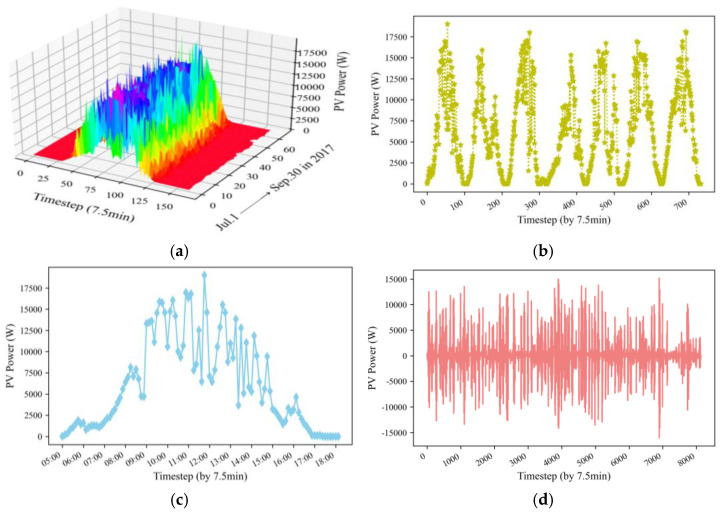
PV power generation data. (**a**) The whole dataset in three dimensions, (**b**) Weekly frequency for the years 1 July 2017–7 July 2017, (**c**) A detailed observation of PV power in 1 July 2017, (**d**) The first different result of the whole dataset.

The diagram of dataset 1 shows a clear demonstration in Figure 4 [39,40]. Figure 4a depicts the entire 7.5 min-ahead historical PV power generation data. Figure 4b plots PV power data from the first week of July 2017. A detailed observation of PV power on 1 July 2017, is shown in Figure 4c. The figures show that the data first rose at noon to reach the peak of power generation and then declined during the day. This trend is repeated every day, and the pattern is similar. Figure 4d depicts the first differential result from dataset 1. As we can see, after the first-order difference, the average value of the data returns to zero, indicating an inevitable stability trend. Table 1 presents the results of the ADF test and Ljung–Box (LB) test. Firstly, we performed the ADF test to check the stability of the PV power time series. The test statistic is less than the critical value of 1% (−3.43), 5% (−2.86), and 10% (−2.57) in the three confidence intervals. The *p*-value is less than 0.05 or equal to zero. Therefore, dataset 1 and dataset 2 are stationary series. Secondly, we used the Ljung–Box (LB) test to determine the randomness of the PV power time series. Each *p*-value of the white noise test is less than 0.05, indicating that the datasets are all nonwhite noise sequences. Thirdly, we performed missing value processing and data standardization. In addition, a fixed-size window slides across the raw PV power dataset [41]. We used the values in the window as the training input vector and the PV power at the next moment as the training target, respectively. During the test, we used the power value in the current sliding window as model input to predict the PV power at the next moment.

### 3.2. Experimental Setup

All programming work is completed on TensorFlow-GPU 2.6.0 and Keras 2.3.1. The hardware configurations are an AMD Radeon (TM) graphics CPU and an NVIDIA GeForce RTX 3050 Ti laptop GPU. The software environment is Python 3.7.0 on the Win11 system.

A three-step comparison is completed in this work. Firstly, we selected several classic machine learning methods, including decision tree, random forest, support vector regression, and multilayer perceptron. Secondly, we explored the effects of the Bayesian optimization algorithm on the LSTM neural network. Finally, we compared the performance of the proposed SSA–BiLSTM method with multiple LSTM extension hybrid models with data decomposition algorithms, including SWT, VMD, and SSA.

Four evaluation metrics, namely mean absolute error (*MAE*), root mean square error (*RMSE*), adjusted coefficient of determination (*Adj-R*^2^), and accuracy (*ACC*), are used in the experiment. Table 2 shows the equation for those criteria. It should be mentioned that the lower the *MAE* and *RMSE* values are, the higher the *Adj-R*^2^ and *ACC* values, and the more accurate the model forecasts.

### 3.3. Hyperparameter Searching

The basic structure of the LSTM model consists of hidden layers, fully connected layers, and output layers in this study. RMSProp is employed to optimize the weights and biases in the LSTM network. The change in parameters may lead to different prediction performances. Hence, a hyperparameter search is performed based on Bayesian optimization, including the number of hidden layer units, the number of fully connected layer units, the learning rate, and the decay. The hyperparameter ranges are listed in Table 3.

New ensemble hyperparameters can be selected with the UCB method. The root mean squared error is calculated as a loss function. The total number of iterations is 20. Through iterations of Bayesian optimization, the best set of hyperparameters will be used to build the final predictive neural network model. Table 4 shows the iterative process of these four hyperparameters in dataset 1. The loss function reaches a minimum at the 14th iteration. The set of hyperparameters will be chosen in the LSTM model to predict the PV power dataset 1.

**Figure 5:**
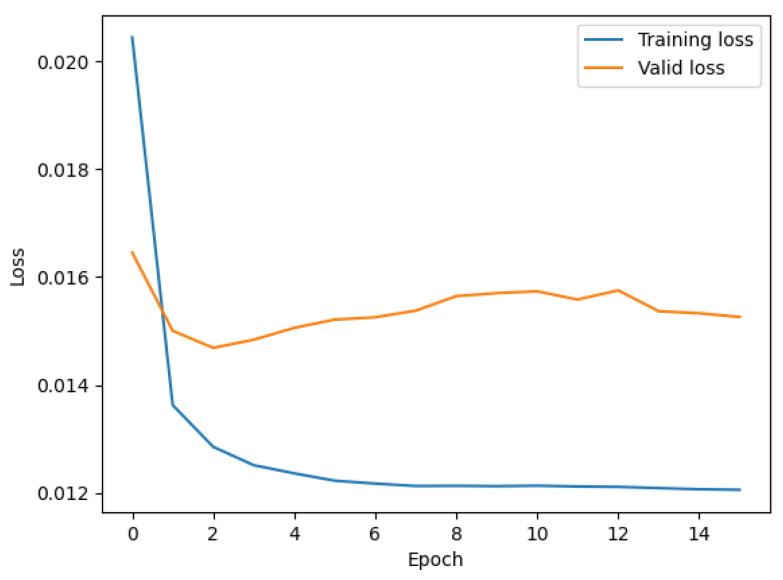
The training and validation loss curves of the BO-LSTM model.

**Figure 6:**
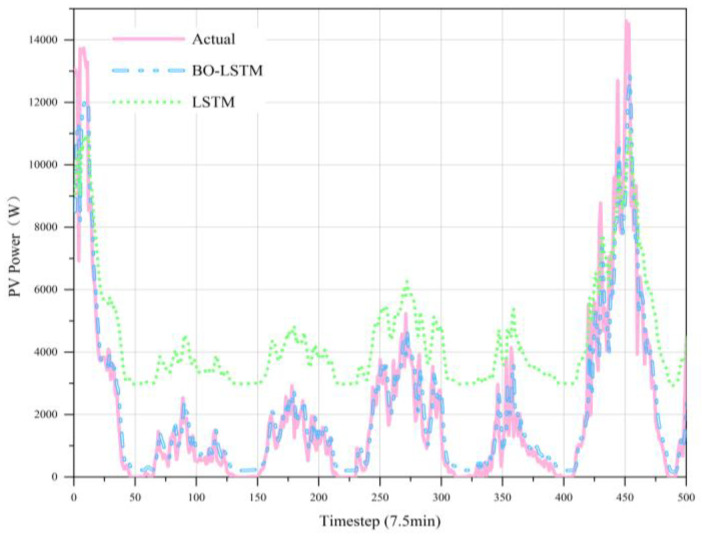
Forecast results of PV power in dataset 1.

The training and valid loss curves of the BO-LSTM model are shown in Figure 5, which show no symptoms of overfitting or underfitting. Figure 6 shows the fitted graph for LSTM and BO-LSTM based on historical data. The case indicates that the BO algorithm in PV power systems significantly affects the prediction effect. This means that the BO-based LSTM neural network forecast model can achieve a higher accuracy due to an efficient way to train the neural networks.

### 3.4. Results of the Experiment

The forecasting results of PV power produced by all the contrast methods with four evaluation metrics are given in Table 5 and Table 6, including *MAE*, *RMSE*, *Adj-R*^2^, and *ACC*. Table 5 reports the results of PV power prediction on dataset 1. These results illustrate that the proposed method outperforms all compared methods in terms of four evaluation metrics. From Table 5, we can see that the proposed method obtains the best results 7.5 min-ahead with values of 3.18 and 4.72 for the *MAE* and *RMSE* metrics, respectively. In addition, the *MAE* of the proposed method is reduced by 199.37% and 111.12% compared with the worst-performing model and reduced by 51.26% and 17.90% compared with the best-performing model, respectively. Similarly, the *RMSE* can obtain an identical conclusion. *Adj-R*^2^ is raised from 0.766 to 0.984 and 0.747 to 0.971, respectively. The *Adj-R*^2^ fitting effect is greatly improved. Similarly, the forecasting accuracy of the proposed algorithm rises sharply. Additionally, it is noted that the *RMSE* and *MAE* increase and the *Adj-R*^2^ and *ACC* values decrease for all the contrast methods from 7.5 min-ahead to 15 min-ahead. The experimental results on dataset 2 are shown in Table 6. Compared with thirteen existing prediction methods, the SSA-BiLSTM model performs best in different timesteps. For example, with 7.5 min-ahead horizon forecasting, the *MAE*, *RMSE*, *Adj-R*^2^, and *ACC* metrics have values of 3.60, 6.42, 0.975, and 74.23%, respectively. The proposed method is the best value among all the compared models. The SWT-NLSTM model places at the second-best position by obtaining the values of 3.93, 7.32, and 0.967 for *MAE*, *RMSE* metrics, and *Adj-R*^2^, respectively. Additionally, the performance of the algorithm is further verified. From one-step to two-step, the amount of error forecast by all methods increases, which means it is more challenging to predict PV power.

In summary, we have compared the machine learning methods, including DTR, RF, SVR, and MLP. The proposed model is compared with the LSTM model, the nested LSTM (NLSTM) model [43], and the bidirectional LSTM (BiLSTM) model [45]. The proposed and compared methods also use data decomposition techniques, namely, SWT [19], VMD [46], and SSA [47]. From the experimental results shown in Table 7, the mean *MAE* of the prediction error of LSTM and its extended model is 11.48, and the *RMSE* is 17.37. For the model using the SWT algorithm, the mean *MAE* of the prediction error is 8.76, and the *RMSE* is 14.18. It improves by 31.05% and 22.50%, respectively. Similarly, using the VMD algorithm model, the year-on-year increases were 18.72% and 24.43%, respectively. Compared to SWT and VMD, DL models combined with SSA perform more accurate predictions and produce less error. For the model using the SSA algorithm, the mean prediction error *MAE* is 6.18, and the *RMSE* is 9.25. Compared with the LSTM class model, it is improved by 85.76% and 87.78%, respectively. The forecast results indicate that the proposed method outperforms the existing compared methods in terms of efficiency and effectiveness.

**Figure 7:**
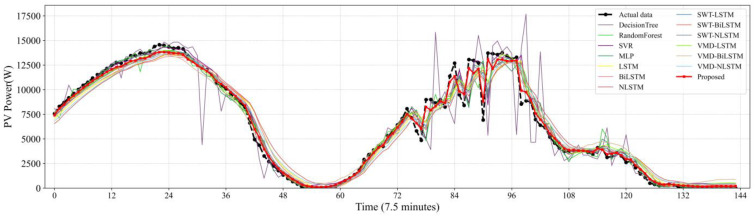
Contrasting the final prediction result with the actual value.

**Figure 8:**
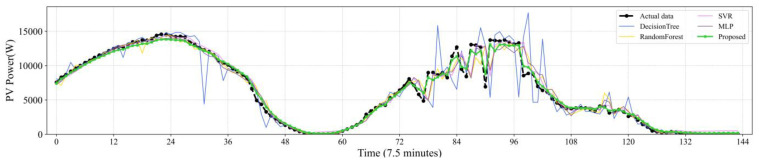
Contrasting the performance evaluation of the proposed model and machine learning models.

**Figure 9:**
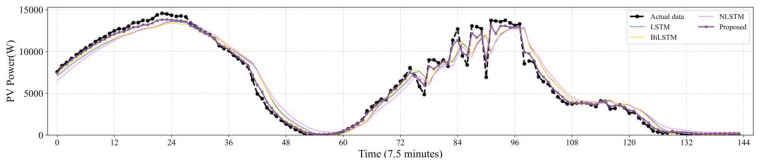
Contrasting the performance evaluation of the proposed model and LSTM and extensions.

**Figure 10:**
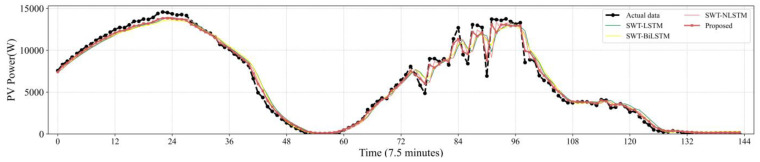
Contrasting the performance evaluation of the proposed model and LSTM networks combining SWT.

**Figure 11:**
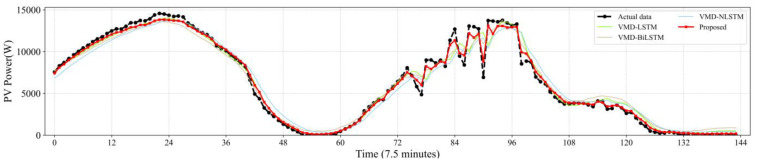
Contrasting the performance evaluation of the proposed model and LSTM networks combining VMD.

Figure 7, Figure 8, Figure 9, Figure 10 and Figure 11 reveal the actual values of the PV power and their forecasted values obtained by the proposed method and other methods. The forecast results of the proposed method and all compared methods of 7.5 min-ahead prediction for dataset 1 are displayed in Figure 7. The predicted and actual values of the proposed model neural network framework better fit not only during the rise or fall of the load but also during the peak and valley of the load. As seen in Figure 8, machine learning algorithms have significant lag effects. Figure 9 shows that the single LSTM model and its extended learning ability are insufficient, and there are obvious prediction errors. In Figure 10, the model adopting VMD to decompose PV power data is less efficient than the proposed model. Figure 11 shows that the SWT has low accuracy in predicting small fluctuations. Some peaks show the opposite trend. As shown in Figure 7 and Figure 11, compared with the proposed SSA, the SWT embedded model is too smooth and less accurate in predicting small fluctuations in PV power data. Some of the peaks have opposite trends, resulting in untimely and insignificant predictions.

**Figure 12:**
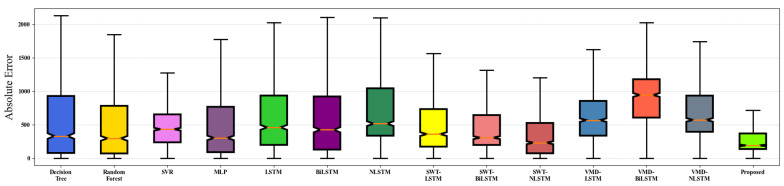
The absolute error of the proposed model and the compared methods.

Table 8 shows the training time for different forecasting algorithms. It is highlighted that the training time for ML models is short. Therefore, these models are not reported. The training time of the proposed DL model is completed in 159 s or less, which has high practicability. Additionally, the boxes of the absolute error of the proposed method and the contrast methods are shown in Figure 12. The proposed method has a lower average absolute error (AE) and narrower distribution. This means that the predicted values are more accurate and reflect the actual value consistently.

## 4. Discussion

From the experimental results, the following information can be obtained:

(1) The lengths of the forecast horizons have negative impacts on the performance of the prediction methods.
(2) The Bayesian optimization algorithm can improve the forecasting accuracy of deep neural networks.
(3) Decomposition-based hybrid methods are superior to most single methods.
(4) Most of the outperformance is achieved with the unique BiLSTM neural network. It obtains the forward and backward characteristics of PV power data. This approach enables the experimental results to be predicted more accurately by providing more thorough feature information. Although NLSTM also improves the prediction accuracy with the SWT decomposition method, the computational time of the NLSTM training process is significantly longer than BiLSTM. Therefore, the BiLSTM model is more suitable for PV power forecasting.
(5) VMD decomposes the original PV power data to obtain modal functions of various frequencies, mainly divided into trend and fluctuation components. However, raw PV power data usually exhibit unstable and irregular properties. VMD is effective at predicting trends but poorly fitted at the cutting edge.
(6) SWT analyses the local variations in the raw data using a local and adaptively long wavelet. The SWT decomposition technique lacks further analysis of high-frequency sub-signals. As a result, models incorporating SWT are insensitive to high-frequency fluctuations.
(7) The PV power record is full of frequent and dramatic fluctuations. The characteristics of the dataset mean that SSA is more suitable for decomposing PV power data. On the one hand, the original signal is decomposed into several sub-signals. Each BiLSTM is used to train the corresponding sub-signal generated by the SSA. The learning sub-signal is conducted separately, increasing DL networks’ attention to every part of the data features. On the other hand, this processing eliminates the influence of data noise and has high noise immunity. The summation of the sub-signal prediction results during the training process is optimized. It can help to mitigate insensitivity to peak prediction issues.

## 5. Conclusions

In this paper, we have proposed a data-driven framework called SSA-BO-BiLSTM to predict PV power. In the architecture of the proposed SSA-BO-BiLSTM, the SSA is used for both denoising and feature extraction; the parallel BiLSTM is designed to predict the corresponding PV power sub-signal. In addition, we applied the Bayesian optimization algorithm to select hyperparameters for the deep BiLSTM architectures. Furthermore, the prediction vectors of the sub-signals are summed to bring about the final forecasting results. The efficiency of SSA-BO-BiLSTM is verified through experiments on real datasets with PV power under 7.5 min-ahead and 15 min-ahead forecasting scenarios. Case studies revealed that the proposed SSA-BO-BiLSTM model outperforms other cutting-edge methods in terms of *MAE*, *RMSE*, *R*^2^, and *ACC* metrics. In future work, we plan to explore a general model for load forecasting for different power stations. Therefore, we intend to securely leverage data from multiple parties with federated learning and transfer learning.

## Figures and Tables

**Table 1:** Statistical information description of the PV power dataset.

Dataset	Maximum (KW)	Mean (KW)	Standard Deviation (KW)	ADF Test Statistic	*p*-Value	LB Test
Dataset 1	20.07	5.62	4.99	−17.46	4.61 × 10^−30^	7.12 × 10^−206^ *
Dataset 2	20.13	4.92	4.86	−24.77	0.0	0.0

* refers to the maximum *p*-value corresponding to the number of delays tested.

**Table 2:** Formulas of the accuracy metrics.

Accuracy Metrics	Formulas
*MAE*	$MAE=\left(\Sigma_{t=1}^{n}\left|X_{t}-\hat{X_{t}}\right|\right)/n$
*RMSE*	$RMSE=\sqrt{\left(\Sigma_{t=1}^{n}{\left[\left|X_{t}-\hat{X_{t}}\right|\right]}^{2}\right)/n}$
*Adj-R* ^2^	$Adj-R^{2}=1-\left(\Sigma_{t=1}^{n}{\left(X_{t}-\hat{X_{t}}\right)}^{2}/\Sigma_{t=1}^{n}{\left(X_{t}-\bar{X_{t}}\right)}^{2}\right)$
*ACC*	$ACC=\left(i_{correct}/i_{all}\right)\times 100$

**Table 3:** The range of hyperparameters.

Parameters in the Bayesian Optimization	Range of Values
Neurons number in LSTM layer	115–525
Neurons number in dense layer	7–65
Learning rate	0.0001–0.001
RMSprop decay	0.01–0.1

**Table 4:** Hyperparameter searching result.

Iteration	Learning Rate	Decay Rate	Neurons Number in LSTM Layer	Neurons Number in Dense Layer	*RMSE*
1	0.000475	0.075	115	25	12.02
2	0.000232	0.018	191	27	11.84
3	0.000457	0.058	287	47	12.36
4	0.000284	0.089	126	46	11.74
5	0.000476	0.060	173	19	12.21
6	0.000336	0.089	124	44	11.71
7	0.000100	0.010	115	36	11.82
8	0.000100	0.010	352	29	11.68
9	0.000100	0.010	483	26	11.64
10	0.000100	0.100	525	16	11.61
11	0.001000	0.010	525	7	26.87
12	0.001000	0.010	164	63	12.26
13	0.000100	0.100	521	40	11.63
**14**	**0.000100**	**0.010**	**525**	**53**	**11.58**

**Table 5:** Performance comparison of different prediction methods based on dataset 1.

Algorithm	7.5 Min-Ahead				15 Min-Ahead			
	*MAE*	*RMSE*	*Adj-* *R* ^2^	*ACC*	*MAE*	*RMSE*	*Adj-* *R* ^2^	*ACC*
Decision Tree	8.91	17.96	0.766	52.55	19.93	36.38	0.747	55.71
Random Forest	6.56	12.35	0.889	53.65	14.88	24.71	0.883	57.92
SVR	6.78	11.90	0.897	53.65	15.00	24.02	0.890	57.11
MLP	6.65	12.31	0.890	55.46	16.63	27.11	0.860	55.91
LSTM [12]	7.63	12.15	0.893	53.25	18.99	26.42	0.867	56.71
BiLSTM [42]	7.21	11.95	0.897	52.95	17.52	25.48	0.876	56.31
NLSTM [43]	8.36	12.70	0.883	56.06	18.18	25.58	0.875	58.52
SWT-LSTM [19]	6.23	10.64	0.918	58.96	15.94	22.51	0.903	61.72
SWT-BiLSTM	5.60	9.41	0.936	64.06	15.06	21.05	0.915	64.13
SWT-NLSTM [13]	4.81	9.51	0.934	64.66	11.13	17.40	0.942	64.73
VMD-LSTM	7.34	10.51	0.920	57.76	15.50	19.95	0.924	65.53
VMD-BiLSTM [44]	9.52	11.12	0.910	69.37	14.52	18.76	0.933	70.74
VMD-NLSTM	7.92	11.29	0.908	59.06	15.38	20.48	0.920	63.73
**Proposed**	**3.18**	**4.72**	**0.984**	**83.58**	**9.44**	**12.29**	**0.971**	**84.77**

**Table 6:** Performance comparison of different prediction methods based on dataset 2.

Algorithm	7.5 Min-Ahead				15 Min-Ahead			
	*MAE*	*RMSE*	*Adj-* *R* ^2^	*ACC*	*MAE*	*RMSE*	*Adj-* *R* ^2^	*ACC*
Decision Tree	6.69	13.63	0.887	55.03	16.57	30.69	0.855	58.32
Random Forest	4.60	8.84	0.952	56.66	10.51	19.12	0.944	66.83
SVR	4.97	8.57	0.955	60.10	9.90	17.61	0.952	66.83
MLP	4.49	8.60	0.955	59.60	10.09	19.22	0.943	64.96
LSTM	6.13	9.96	0.940	62.23	14.06	21.59	0.928	68.21
BiLSTM	5.76	9.69	0.943	62.16	13.14	20.97	0.932	67.46
NLSTM	6.78	10.64	0.931	60.54	13.99	21.31	0.930	68.09
SWT-LSTM	4.85	8.56	0.955	64.35	11.79	19.51	0.941	71.71
SWT-BiLSTM	4.85	8.09	0.960	67.10	12.21	19.24	0.943	72.59
SWT-NLSTM	3.93	7.32	0.967	65.98	8.73	16.89	0.956	72.72
VMD-LSTM	4.74	8.44	0.957	63.73	10.24	17.61	0.952	71.71
VMD-BiLSTM	5.57	8.01	0.961	67.67	9.13	15.16	0.965	74.59
VMD-NLSTM	6.17	9.49	0.945	63.29	9.98	16.69	0.957	72.72
**Proposed**	**3.60**	**6.42**	**0.975**	**74.23**	**8.51**	**13.57**	**0.972**	**78.22**

**Table 7:** The mean *MAE* and *RMSE* of various methods.

Algorithm	*MAE*	*RMSE*
LSTM and extensions	11.48	17.37
LSTM networks combining SWT	8.76	14.18
LSTM networks combining VMD	9.67	13.96
**Proposed**	**6.18**	**9.25**

**Table 8:** Training time in seconds for different forecasting models.

Model	Training Time			
	1#7.5 *	1#15	2#7.5	2#15
LSTM	19	11	27	22
BiLSTM	28	15	48	35
NLSTM	110	55	175	88
SWT-LSTM	66	35	110	56
SWT-BiLSTM	108	56	161	83
SWT-NLSTM	438	218	905	602
VMD-LSTM	67	35	166	82
VMD-BiLSTM	107	56	263	131
VMD-NLSTM	433	218	1072	593
**Proposed**	**107**	**56**	**159**	**132**

* “1#7.5” refers to dataset 1 7.5 min-ahead.

## Data Availability

The required datasets for the experiment can be obtained for free from https://dx.doi.org/10.21227/9hje-dz22 aeecssed on 17 May 2022.
